# Multiple Immunosuppressive Effects of CpG-c41 on Intracellular TLR-Mediated Inflammation

**DOI:** 10.1155/2017/6541729

**Published:** 2017-04-30

**Authors:** Wancheng Liu, Xuejiao Yang, Ning Wang, Shijun Fan, Yuanfeng Zhu, Xinchuan Zheng, Yan Li

**Affiliations:** Medical Research Center, Southwest Hospital, Third Military Medical University, Chongqing 400038, China

## Abstract

A growing body of literature suggests that most chronic autoimmune diseases are associated with inappropriate inflammation mediated by Toll-like receptor (TLR) 3, TLR7/8, or TLR9. Therefore, research into blocking TLR activation to treat these disorders has become a hot topic. Here, we report the immunomodulatory properties of a nonstimulatory CpG-containing oligodeoxynucleotide (CpG-ODN), CpG-c41, which had previously only been known as a TLR9 antagonist. In this study, we found that both in vitro and in vivo CpG-c41 decreased levels of various proinflammatory factors that were induced by single activation or coactivation of intracellular TLRs, but not membrane-bound TLRs, no matter what downstream signal pathways the TLRs depend on. Moreover, CpG-c41 attenuated excessive inflammation in the imiquimod-induced psoriasis-like mouse model of skin inflammation by suppressing immune cell infiltration and release of inflammatory factors. We also found evidence that the immunosuppressive effects of CpG-c41 on other intracellular TLRs are mediated by a TLR9-independent mechanism. These results suggest that CpG-c41 acts as an upstream of signaling cascades, perhaps on the processes of ligand internalization and transfer. Taken together, these results suggest that CpG-c41 disrupts various aspects of intracellular TLR activation and provides a deeper insight into the regulation of innate immunity.

## 1. Introduction

The complex mechanisms driving the pathogenesis of autoimmune diseases remain poorly understood. The drugs currently in clinical use cannot effectively eliminate autoimmune diseases and may cause side effects. In recent years, an increasing number of studies have shown that innate immune disorders are closely related to autoimmune diseases [1]. The pattern recognition receptors (PRRs) of the innate immune system are able to recognize pathogen-associated molecular patterns (PAMPs), which trigger relevant signal transmission leading to inflammatory responses. Unfortunately, excessive inflammation can induce autoimmune diseases, such as psoriasis, systemic lupus erythematosus, and rheumatoid arthritis [2–5]. Therefore, identification of new therapeutic targets to ameliorate autoimmune pathogenesis has become a research priority.

Toll-like receptors (TLRs) are a family of proteins expressed in dendritic cells (DCs) and macrophages, which constitute the first line of immunological defense against a variety of pathogens [6, 7]. TLRs recognize specific PAMPs: TLR3 and TLR7/8 recognize double-stranded and single-stranded (ss) RNA, respectively, and TLR9 recognizes unmethylated CpG-DNA [8–12]. The receptors utilize various downstream signaling cascades; for example, TLR3 depends on the TRIF pathway and TLR7 on the MyD88 pathway. Nevertheless, activation through different TLRs induces similar proinflammatory responses, characterized by release of factors such as TNF-*α* and IL-6 [13]. TLR activation can result in the formation of the Nod-like receptor 3 (NLRP3) inflammasome [14] and promote the release of IL-1*β* and IL-18, which are involved in many diseases [15, 16].

The TLRs and their associated pathways constitute an interlaced network, which makes it difficult to identify rational therapeutic targets. Moreover, excessive inflammation is often caused by multiple PAMPs [17–19]. Thus, the coactivation of numerous TLRs adds to the complexity. Current drugs nonselectively target the terminal process and inhibit the resulting proinflammatory factors. Antibodies against TNF-*α*, IL-17, and IL-23 have all been used to treat psoriasis. Although this therapeutic strategy has shown some promise, it is also associated with a higher risk of serious infections [20, 21]. By contrast, targeting upstream processes could decrease side effects and serve as an optimal therapeutic strategy. Unfortunately, no rational drug target has yet been identified.

Here, we report new findings on a nonstimulatory CpG-containing oligodeoxynucleotide (CpG-ODN), previously known only as a TLR9 antagonist, CpG-c41 [22]. We present multiple immunosuppressive effects of CpG-c41 on intracellular TLR-mediated activity. These results indicate that it may be possible to develop drugs that target upstream processes in innate immune cells to treat autoimmune diseases.

## 2. Materials and Methods

### 2.1. Animals

Wide-type (WT) female BALB/c mice (8–10 weeks) were purchased from HFK Bioscience (Beijing, China), and TLR9^−/−^ C57BL/6 mice (6–8 weeks) were obtained from the Chinese Academy of Inspection and Quarantine (Beijing, China). Mice were housed in the Experimental Animal Platform of the Medical Research Center at the Third Military Medical University and kept under specific-pathogen-free conditions with free access to food and water. All animal experiments were performed in accordance with the National and Institutional Guidelines for Animal Care and Use and approved by the Institutional Animal Ethics Committee of the Third Military Medical University.

### 2.2. Cell Culture

The mouse RAW264.7 macrophage cell line was cultured in Dulbecco's modified Eagle's medium (DMEM) (Gibco, USA), and human monocytic THP-1 cells were grown in RPMI-1640 (Gibco, USA).

We differentiated mouse bone marrow cells into bone marrow-derived macrophages (BMDMs) and bone marrow-derived dendritic cells (BMDCs). Briefly, bone marrow cells were flushed from mouse femurs and tibiae and then maintained in lineage-specific differentiation media. BMDMs were maintained in macrophage differentiation medium (DMEM with 40 ng/ml M-CSF (Sigma-Aldrich)) for 5 days. Approximately 96–99% of the cells from the BMDM cultures were F4/80^+^ assessed by confocal image analysis. BMDCs were maintained in DC differentiation medium (RPMI-1640 with 20 ng/ml GM-CSF and 10 ng/ml IL-4 (Sigma-Aldrich)) for 5 days. Approximately 70% of the cells from the BMDC cultures were CD11C^+^.

All media were supplemented with 10% fetal bovine serum (Hyclone Laboratories), 2 mM L-glutamine, 100 U/ml penicillin, and 100 *μ*g/ml streptomycin.

### 2.3. TLR Agonists and CpG-ODNs

We purchased zymosan (TLR2 agonist) and lipopolysaccharide (LPS, TLR4 agonist) from Sigma-Aldrich and polyI:C (TLR3 agonist), imiquimod (TLR7 agonist), and R848 (TLR7/8 agonist) from InvivoGen. We purchased ssRNA120 (TLR7/8 agonist) from Sangon Biotech (China). We mixed ssRNA120 with DOTAP (N-[1-(2,3-dioleoyloxy)propyl]-N,N,N-trimethylammonium methyl sulfate) liposomal transfection reagent (Roche) before use.

Single-stranded CpG-ODNs were synthesized and purified by Sangon Biotech (China). The CpG-ODNs were used in this study, including CpG-ODN 1826 (CpG-1826, 5′-TCCATGACGTTCCTGACGTT-3′) and CpG-c41 (5′-TGGCGCGCACCCACGGCCTG-3′).

### 2.4. ELISA

We measured the concentration of cytokines TNF-*α*, IL-6, IL-1*β*, and IL-23 in cell culture supernatants and cytokines TNF-*α*, IL-6, IFN-*α*, and IL-12/23p40 in mouse sera by ELISA, according to the manufacturer's instructions (eBioscience, USA).

### 2.5. Western Blot (WB) Analysis

Total proteins were extracted, and the protein concentration was determined using a bicinchoninic acid (BCA) assay kit (Beyotime Biotechnology). Sample proteins were separated by SDS-PAGE and then incubated with primary antibodies against NLRP3 (2 *μ*g/ml, R&D systems), caspase-1 (1 : 1000, Abcam), or tubulin (1 : 1000, Beyotime Biotechnology) at 4°C overnight, followed by horseradish peroxidase-labeled IgG (H + L) (1 : 2000, Beyotime Biotechnology). We normalized the levels of our target proteins to tubulin. The membranes were scanned with the ChemiDoc™ XRS+ system (Bio-Rad, USA).

### 2.6. Induction and Treatment of Disease

We induced psoriasis with commercially available Aldara cream (5% imiquimod (IMQ)) (3M Pharmaceuticals, UK). Female BALB/c mice (8–10 weeks) were divided into placebo and treatment groups. We administered phosphate buffer saline (PBS) to the placebo group and CpG-c41 (320 *μ*g/20 g) to the treatment group by subcutaneous injection at multiple points (total, 100 *μ*l/mouse); we topically applied 45 mg of IMQ cream to the shaved back skin of both groups once per day. Induction of disease was performed over 6 consecutive days.

### 2.7. Psoriasis Area and Severity Index (PASI)

PASI was recorded daily. Three parameters (thickness, erythema, and scaling) were evaluated and scored independently on a scale from 0 to 4 (0, none; 1, slight; 2, moderate; 3, marked; and 4, very marked). The cumulative score was the sum of the three parameters, ranging from 0 to 12.

### 2.8. Histology and Immunofluorescence

For histological assessment, samples of dorsal skin from the disease model (day 7) were fixed in 10% formalin for ≥24 h at 23°C and embedded in paraffin. Deparaffinized 5 *μ*m sections were stained with hematoxylin erythrosine saffron and assessed by light microscopy.

For histological immunofluorescence assessment, we prepared 5 *μ*m frozen sections of dorsal skin from 24 hours, 72 hours, and 7 days after disease induction. Monoclonal antibody to F4/80 (Alexa Fluor 488, 1 : 150), primary antibodies to IL-23p19 (1 : 200) and CD3 (1 : 150), and Cy3-labeled goat anti-rabbit IgG (H + L) secondary antibody (1 : 1000) were used according to the manufacturer's instructions (Abcam).

### 2.9. Statistical Analysis

Data are expressed as mean ± SEM and analyzed using the independent sample *t*-test. Where *P* values were <0.05, differences were considered statistically significant.

## 3. Results

### 3.1. CpG-c41 Suppresses Intracellular TLR-Induced Inflammation

**Figure 1:**
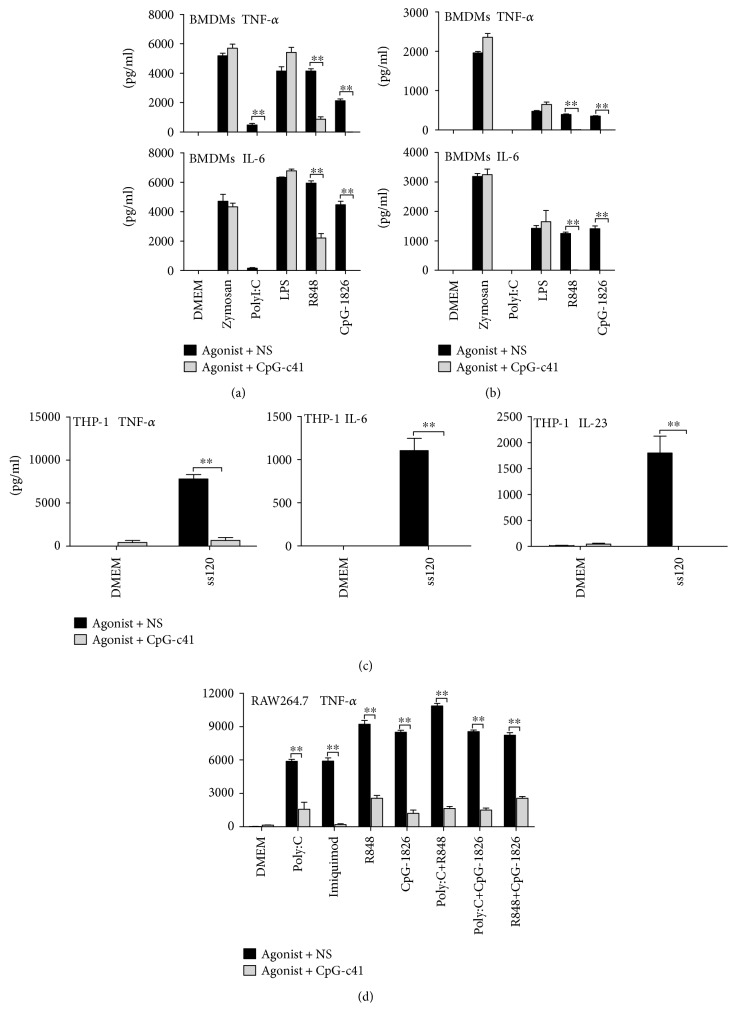
CpG-c41 inhibits cytokine secretion driven by intracellular TLR activation in vitro. Effects of CpG-c41 on cytokine secretion induced by TLR activation in WT BMDMs (a) and BMDCs (b), TLR8 activation in THP-1 cells (c), and dual TLR activation in RAW264.7 cells (d). All cells were seeded into 96-well tissue culture plates at 5 × 10^5^ cells/200 *μ*l/well in the presence or absence of CpG-c41 (4 *μ*M) for 24 hours and stimulated as indicated: zymosan (200 *μ*g/ml), polyI:C (100 *μ*g/ml), LPS (100 ng/ml), imiquimod (2 *μ*g/ml), ssRNA120 (30 *μ*g/ml) mixed with DOTAP, R848 (0.2 *μ*g/ml), and CpG-1826 (2 *μ*M). Cytokines in cell-free culture supernatants were determined by ELISA. ^∗∗^*P* < 0.01. Bars represent mean ± SEM (*n* = 4). NS, normal saline.

We investigated the effects of nonstimulatory CpG-c41 on the activation of TLRs in murine BMDMs and BMDCs. CpG-c41 suppressed the secretion of various proinflammatory factors induced by TLR3, TLR7, or TLR9 agonists (polyI:C, R848, or CpG-1826, respectively) but not those induced by TLR2 or TLR4 agonists (zymosan or LPS) (Figures 1(a) and 1(b)).

We also observed the effects of CpG-c41 on TLR8 activation in the human monocytic cell line THP-1. We found that CpG-c41 also significantly suppressed TLR8 activation induced by ssRNA120 (TLR7/8 agonist) [23] (Figure 1(c)).

TLR3, TLR7/8, and TLR9 are intracellular receptors, and TLR2 and TLR4 are cell membrane receptors. Therefore, these data indicate that CpG-c41 selectively suppresses intracellular, but not cell membrane, TLRs.

Moreover, we investigated the effects of CpG-c41 on RAW264.7 cells in which two intracellular TLRs were stimulated simultaneously. Again, CpG-c41 significantly decreased proinflammatory factor release (Figure 1(d)). Thus, CpG-c41 appeared to have an immunosuppressive effect on TLR coactivation.

**Figure 2:**
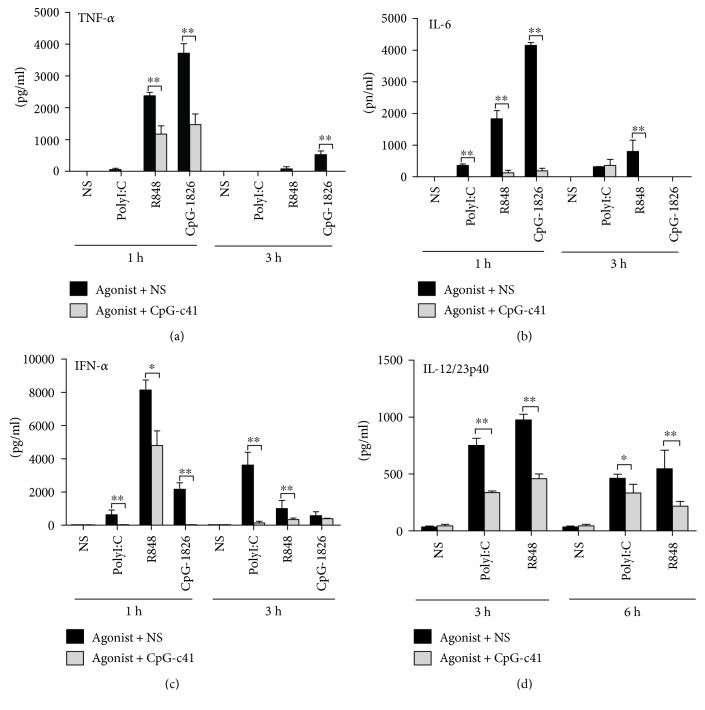
CpG-c41 alters TLR activation-induced cytokine secretion in vivo. WT mice received intraperitoneal injection of polyI:C (40 *μ*g/20 g), R848 (10 *μ*g/20 g), or CpG-1826 (160 *μ*g/20 g), as indicated. They also received CpG-c41 (320 *μ*g/20 g) or normal saline (NS) by tail vein injection. Cytokines in sera from the indicated time points were determined by ELISA. ^∗∗^*P* < 0.01. Bars represent mean ± SEM (*n* = 4).

We then studied the effects of CpG-c41 in vivo. BALB/c serum TNF-*α*, IL-6, and IFN-*α* levels were elevated an hour after the treatment with TLR agonists and were significantly decreased 3 hours after the treatment (Figures 2(a), 2(b), and 2(c)). Serum IL-12/23p40 levels were elevated in the third hour and then significantly decreased in the sixth hour after the treatment (Figure 2(d)). Treatment with CpG-c41 decreased levels of serum cytokines at each time point.

### 3.2. CpG-c41 Inhibits TLR-Mediated Inflammasome Formation and Activation

TLR activation not only induces proinflammatory factor release but also promotes formation of the NLRP3 inflammasome [24]. We investigated the effects of CpG-c41 on the basic elements and downstream effector molecules of the inflammasome.

**Figure 3:**
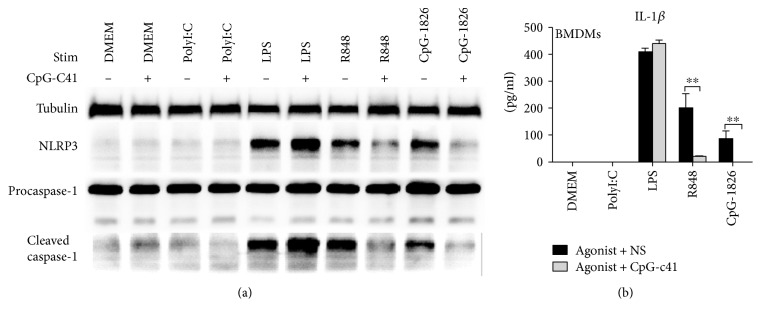
CpG-c41 affects inflammasome formation and activation. RAW264.7 cells were seeded into 6-well culture plates at 3 × 10^6^/ml/well and BMDMs were seeded into 96-well culture plates at 5 × 10^5^/200 *μ*l/well. Cells were stimulated with polyI:C (100 *μ*g/ml), LPS (100 ng/ml), R848 (1 *μ*g/ml), or CpG-1826 (3 *μ*M) in the presence or absence of CpG-c41 (8 *μ*M) for 4 hours. Cells were then stimulated with ATP (5 mM) for 30 min. (a) NLRP3 and caspase-1 protein expression in RAW264.7 cells were detected by WB, with tubulin as an internal control. Representative data from 1 of 3 independent experiments are shown. (b) IL-1*β* production in cell-free supernatants from BMDM cultures was measured by ELISA. ^∗∗^*P* < 0.01. Bars represent mean ± SEM (*n* = 4).

We found that LPS, R848, and CpG-1826 increased the expression of NLRP3, induced the cleavage of caspase-1 (Figure 3(a)), and promoted the secretion of IL-1*β* (Figure 3(b)). CpG-c41 interfered with the inflammasome activation induced by R848 and CpG-1826. It decreased the levels of NLRP3 and cleaved caspase-1 and significantly reduced IL-1*β* release (Figure 3). Interestingly, we did not detect polyI:C-induced inflammasome activation, in contrast to a previous report [25].

### 3.3. TLR9-Independent Immunosuppressive Effects of CpG-c41

**Figure 4:**
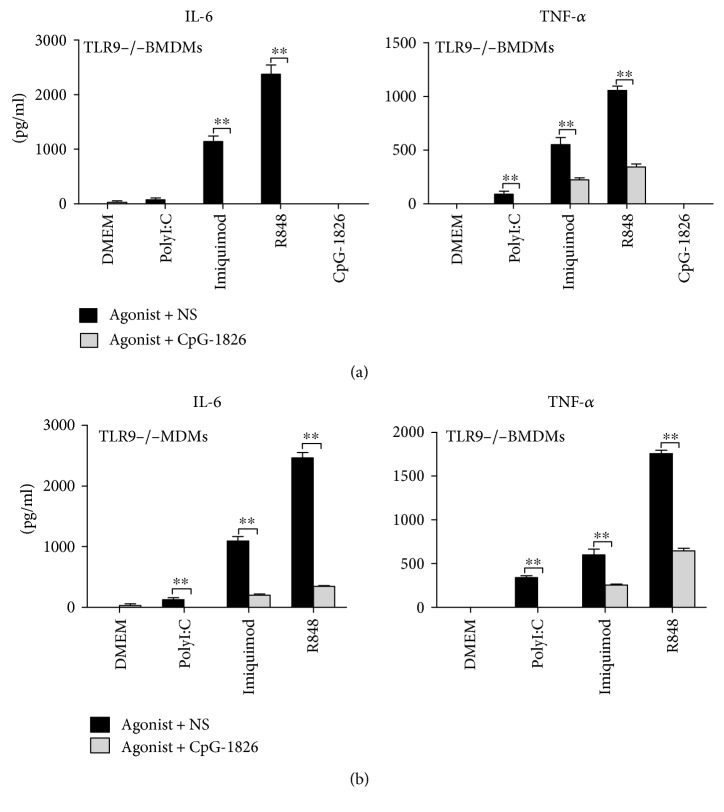
CpG-c41 and CpG-1826 inhibit cytokine secretion by TLR9^−/−^ BMDMs. TLR9^−/−^ BMDMs were seeded into 96-well culture plates at 5 × 10^5^/200 *μ*l/well. (a) Cells were stimulated with polyI:C (100 *μ*g/ml), imiquimod (2 *μ*g/ml), R848 (0.2 *μ*g/ml), and CpG-1826 (2 *μ*M) in the presence or absence of CpG-c41 (4 *μ*M) for 24 hours. (b) Cells were stimulated with polyI:C (100 *μ*g/ml), imiquimod (2 *μ*g/ml), and R848 (0.2 *μ*g/ml) in the presence or absence of CpG-1826 (4 *μ*M) for 24 hours. Cytokines in cell-free culture supernatants were determined by ELISA. ^∗∗^*P* < 0.01. Bars represent mean ± SEM (*n* = 4).

TLR9 specifically recognizes CpG-ODNs, and the nonstimulatory CpG-c41 molecule was previously known only as a TLR9 antagonist [22]. Therefore, we investigated if the immunosuppressive effects of CpG-c41 on cytokine secretion downstream of other TLRs were related to TLR9-mediated crosstalk. We repeated the in vitro experiments using TLR9^−/−^ BMDMs. We found that CpG-1826, a TLR9 agonist, lost its immunostimulatory effect in TLR9^−/−^ BMDMs; however, TLR3 and TLR7 could be activated normally by their ligands. Interestingly, nonstimulatory CpG-c41 was still able to significantly suppress the releases of TNF-*α* and IL-6 induced by TLR3 and TLR7 activation in the TLR9^−/−^ BMDMs (Figure 4(a)). These findings indicate that the immunosuppressive effects of CpG-c41 on TLR3 and TLR7 do not require interaction with TLR9.

Although the TLR9 agonist CpG-1826 lost its immunostimulatory function in TLR9^−/−^ BMDMs, we observed that it could significantly suppress the release of TNF-*α* and IL-6 induced by TLR3 and TLR7 stimulation (Figure 4(b)). These results suggest that CpG-ODNs might inhibit TLR3 and TLR7 activation regardless of their immunostimulatory properties in relation to TLR9.

### 3.4. CpG-c41 Attenuates IMQ-Induced Psoriasis-Like Inflammation In Vivo

**Figure 5:**
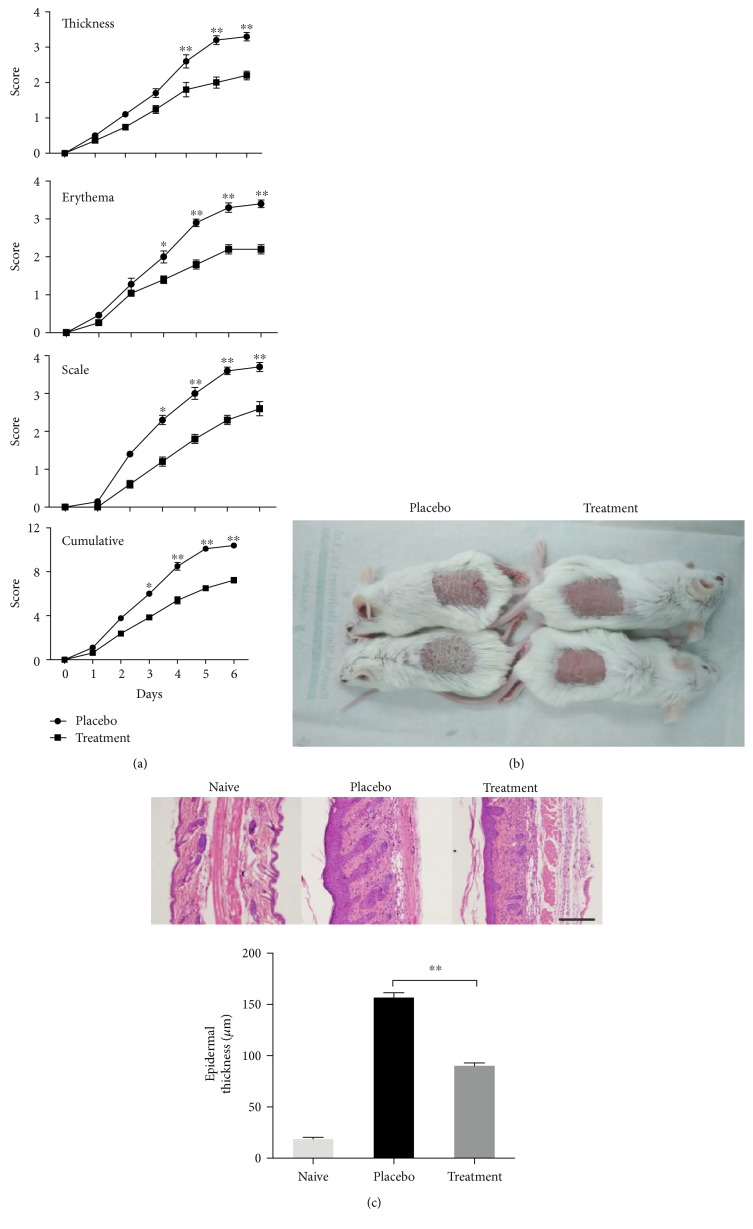
CpG-c41 reduces damage from IMQ-induced psoriasis-like disease. (a) PASI scoring of mice with the IMQ-induced psoriasis-like skin condition receiving placebo or CpG-c41 treatment. (b) Appearance of affected skin on day 6. (c) Histological staining of skin from day 7. Scale bars represent 200 *μ*m. Representative data from 1 of 3 independent experiments are shown. ^∗^*P* < 0.05, ^∗∗^*P* < 0.01. Graphs show mean ± SEM (*n* = 3). Treat, treatment.

Studies have increasingly shown that PAMPs are the precipitating factor for psoriasis, and a psoriasis-like animal model has been developed [26, 27]. We investigated the effects of CpG-c41 in this model. The PASI scores showed that CpG-c41 treatment significantly decreased IMQ-induced skin injury over the course of 6 consecutive days of treatment (Figure 5(a)). In contrast to the skin of the placebo group, the treated skin was relatively smooth with little scaling, lighter erythema, and reduced thickness at day 6 (Figure 5(b)). Pathological analysis showed that papillary hyperplasia was reduced, and the condition of the stratum spinosum and the parakeratosis were improved in the treatment group (Figure 5(c)). These findings indicate that CpG-c41 can attenuate IMQ-induced psoriasis-like inflammation.

**Figure 6:**
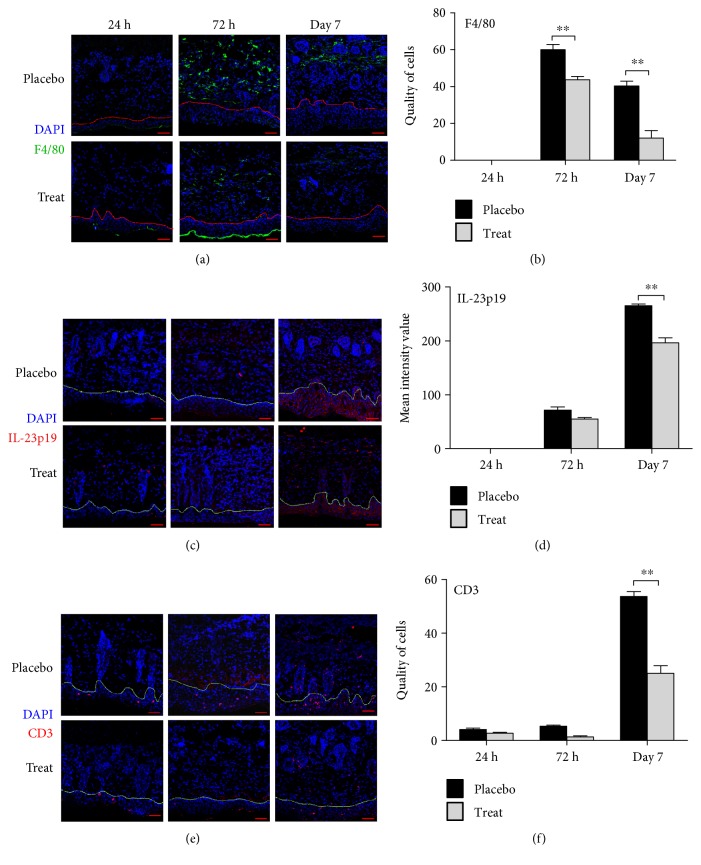
Analysis of inflammatory infiltrates in the affected skin of mice with a psoriasis-like disease by laser confocal microscopy. (a1) Anti-mouse F4/80^+^ (green) and DAPI (blue); (a2) mean quantity of cells per square area. (b1) Anti-mouse IL-23p19 (red) and DAPI (blue); (b2) mean intensity value per square area. (c1) Anti-mouse CD3^+^ (red) and DAPI (blue); (c2) mean quantity of cells per square area. A representative image is given from each of three independent experiments. Scale bars represent 50 *μ*m. ^∗∗^*P* < 0.01. Bars represent mean ± SEM (*n* = 3).

In the pathogenesis of psoriasis, the IL-23/IL-17 axis is believed to play a key role in linking the innate and adaptive immune responses [28]. Thus, we assessed inflammatory infiltrates into IMQ-damaged skin using immunofluorescence microscopy. We observed peak F4/80^+^ macrophage infiltration in the placebo group on day 3, with significantly lower infiltration on day 7 (Figures 6(a1) and 6(a2)). We first detected IL-23p19 on day 3 and found increased expression in the epidermal layer on day 7 (Figures 6(b1) and 6(b2)). Likewise, the distribution of T cells was normal in the skin at day 3, but T cell infiltration increased in the epidermis and dermis on day 7 (Figures 6(c1) and 6(c2)). By contrast, in the treatment group, CpG-c41 reduced macrophage infiltration, decreased IL-23p19 release, and attenuated T cell infiltration.

## 4. Discussion

Researchers have sought to overcome chronic autoimmune diseases for many years. Recent studies have suggested that excessive TLR-mediated inflammation correlates with the occurrence and progression of these diseases [29–31]. In particular, various chronic autoimmune diseases are closely associated with the activation of intracellular TLRs (TLR3, TLR7/8, and TLR9) [32–34]. However, due to the complexity of the TLR signaling network, there have been no breakthroughs in the identification of therapeutic targets thus far.

In this study, we investigated the effects of CpG-c41 on innate immune cells. Both in vitro and in vivo CpG-c41 significantly reduced the secretion of various inflammatory cytokines induced by individual activation or coactivation of intracellular TLRs. It also attenuated inflammatory infiltrates in an IMQ-induced animal model of psoriasis by suppressing macrophage activation. Taken together, these results illustrate the multiple immunosuppressive effects of CpG-c41 on inflammation mediated by various intracellular TLRs.

This study expands our understanding of innate immunity. The members of the TLR family have unique structural features that recognize specific PAMPs, and each family member may be characterized by the signaling pathways it uses to promote inflammatory responses. From another point of view, the TLRs could be classified according to their distribution in cells. Unlike cell membrane TLRs, which undergo direct activation, intracellular TLRs require additional steps to initiate recognition, including ligand uptake and receptor circulation [35]. This study demonstrates that CpG-c41 selectively suppresses the activation of intracellular TLRs. It emphasizes the functional significance of TLR distribution, which provides a new strategy for controlling excessive inflammation by targeting TLRs based on their locations.

Intracellular TLRs generally use distinct downstream signaling cascades; TLR3 signals through the TRIF pathway, while TLR7 and TLR9 signal through the MyD88 pathway. CpG-c41 mediates the same immunosuppressive effects on these TLRs, suggesting that the mechanism of suppression is not related to downstream signaling cascades. Although crosstalk is a common phenomenon due to intersection of the different signaling pathways, this study indicates that the immunosuppressive effects of CpG-c41 on other intracellular TLRs are not dependent on crosstalk with TLR9. Taken together, these findings suggest that CpG-c41 could mediate its suppressive effects by acting on the processes of ligand internalization or transfer, upstream of the signaling cascades.

TLR9 is known to specifically recognize CpG-ODNs and trigger proinflammatory responses. More recent studies have found that many CpG-ODNs do not have immunostimulatory properties; in fact, only some CpG-ODNs block the activation of TLR9 [18, 36]. As we previously reported, CpG-c41, which we screened from a large collection of nonimmunostimulatory CpG-ODNs, has a special sequence structure and an outstanding capacity to suppress TLR9 activation [22]. In this study, we discovered additional evidence that CpG-c41 has multiple immunosuppressive effects. Interestingly, we found that the TLR9 agonist CpG-1826 had similar immunosuppressive effects on TLR3 and TLR7, even when its immunostimulatory function was lost in TLR9^−/−^ cells. Unlike TLR9, the other intracellular TLRs are inhibited by the above CpG-ODNs through an alternative mechanism. Moreover, although both TLR7 and TLR9 depend on the MyD88 pathway, CpG-1826 had immunosuppressive effects in TLR7-replete TLR9^−/−^ conditions. These findings support the interpretation that CpG-ODN-mediated immunosuppression is unrelated to the downstream signaling cascade.

On the other hand, the sequence structures of CpG-ODNs are thought to contribute to their immune characteristics, but, so far, no pattern in the sequences of functional CpG-ODNs has been found. Thus, the relationship between sequence structure and function remains ambiguous. Unlike CpG-c41, some other immunosuppressive CpG-ODNs only inhibit a subset of the intracellular TLRs [36, 37]. By contrast, CpG-c41 has dramatic effects on innate immunity.

In comparison with currently available drugs, CpG-c41 would have several advantages. First, it selectively inhibits the activation of all intracellular, but not cell membrane-bound, TLRs. Second, by blocking upstream events, it could simultaneously suppress multiple proinflammatory factors. Finally, its nonstimulatory nature would not negatively affect the normal immune response, but it could suppress excessive, abnormal inflammation to help patients through the acute phase of disease.

## 5. Conclusion

This study demonstrates the immunosuppressive effects of CpG-c41 on inflammation mediated by various intracellular TLRs, upstream of signaling cascades, and provides a potential approach to regulate innate immunity without targeting downstream signaling cascades.
